# Investigation of the Theoretical Model of Nano-Coolant Thermal Conductivity Suitable for Proton Exchange Membrane Fuel Cells

**DOI:** 10.3390/nano14211710

**Published:** 2024-10-26

**Authors:** Qi Tao, Boao Fu, Fei Zhong

**Affiliations:** 1 School of Mechanical Engineering, Hubei University of Technology, Wuhan 430070, China; taoqi@hbut.edu.cn (Q.T.); 15797335031@163.com (B.F.); 2 Hubei Key Laboratory of Modern Manufacture Quality Engineering, School of Mechanical Engineering, Hubei University of Technology, Wuhan 430068, China

**Keywords:** thermal conductivity, nano-coolant, nanolayer, thickness, PEMFCs

## Abstract

The fuel cell vehicle is one of the essential directions for developing new energy vehicles. But heat dissipation is a critical technical difficulty that needs to be solved urgently. Nano-coolant is a promising coolant that can potentially replace the existing coolant of a fuel cell. However, its thermal conductivity has a significant impact on heat dissipation performance, which is closely related to nanoparticles’ thermal conductivity, nanoparticles’ volume fraction, and the nano-coolant temperature. Many scholars have created the thermal conductivity models for nano-coolants to explore the mechanism of nano-coolants’ thermal conductivity. At present, there is no unified opinion on the mechanism of the micro thermal conductivity of the nano-coolant. Hence, this paper proposed a novel model to predict the thermal conductivity of ethylene glycol/deionized water-based nano-coolants. A corrected model was designed based on the Hamilton & Crosser model and nanolayer theory. Finally, a new theoretical model of nano-coolant thermal conductivity suitable for fuel cell vehicles was constructed based on the base fluid’s experimental data.

## 1. Introduction

### 1.1. Background Description

**Figure 1:**
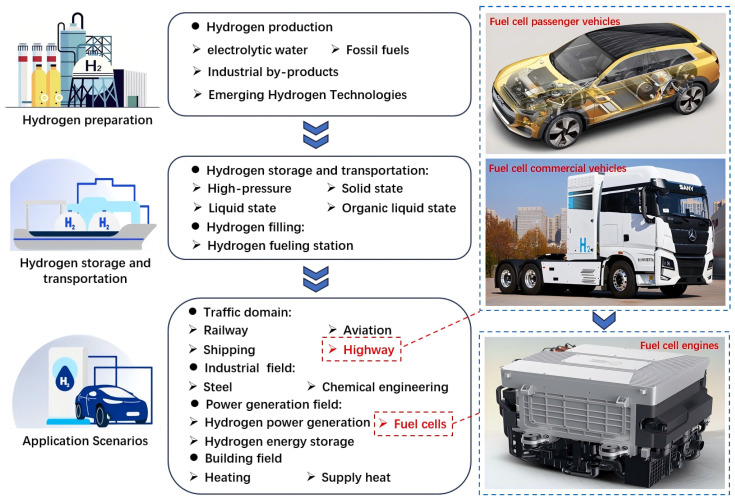
Hydrogen preparation, storage, transportation, and application scenarios.

A fuel cell can convert chemical energy into electrical energy based on the electrochemical reaction of hydrogen and oxygen, which can be divided into a Phosphoric Acid Fuel Cell (PAFC), Solid Oxide Fuel Cell (SOFC), Molten Carbonate Fuel Cell (MCFC), Solid Oxide Fuel Cell (SOFC), Alkaline Fuel Cell (AFC), and Proton Exchange Membrane Fuel Cell (PEMFC) [1]. Among them, the PEMFC has been deemed one of the most suitable energy sources for fuel cell vehicles, which has high efficiency and low pollution [2]. Fuel cell vehicles include ordinary cars, heavy trucks, public transport, cold chain logistics, non-road mobile machinery, etc. High-power fuel cells are mainly used in heavy trucks and public transport, in which problems of large heat dissipation and high heat dissipation energy consumption exist [3]. This can be seen in Figure 1. Due to the space limitations inside the engine, the radiator dimension cannot be increased. The increasing rotation speed of the pump and fan leads to increased heat dissipation energy consumption. An efficient thermal management system can avoid spontaneous combustion or even the explosion of fuel cell vehicles in extreme conditions [4].

**Figure 2:**
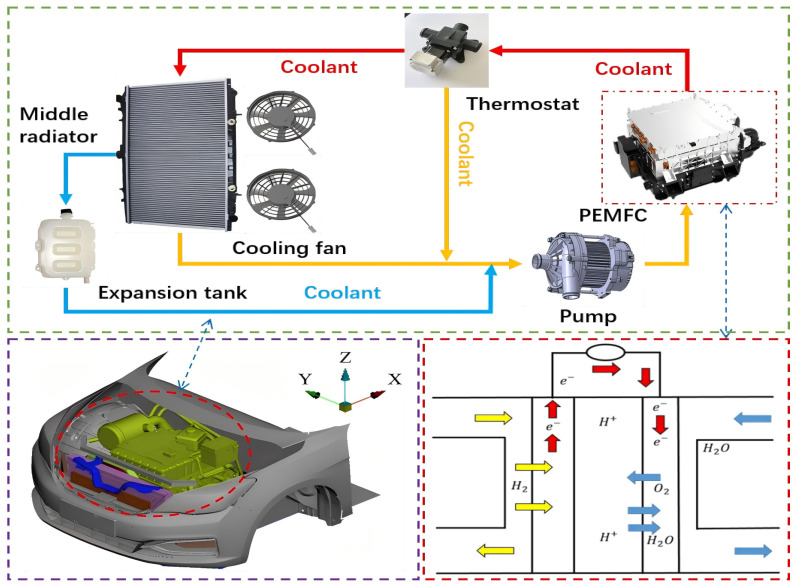
Thermal management system of PEMFCs in fuel cell vehicles.

Establishing a thermal management system of fuel cell vehicles aims to ensure the safe and stable operation of the heat-producing components under different working conditions and to control the circulating water flow and temperature through reasonable heat dissipation methods. The components of a thermal management system of PEMFCs include the PEMFC, pump, radiator, expansion tank, thermostat, etc. This can be seen in Figure 2.

A thermal management system is crucial to increase the durability and efficiency of fuel cells [5]. Due to its importance, many scholars delved into the PEMFC’s thermal management system in recent years. For example, Zhu [6] investigated and proposed cooling strategies of PEMFC’s thermal management system. After that, Zhu analyzed its heat dissipation requirements of fuel cell vehicles in a high-temperature environment. Huang [7] reviewed the heat transfer mechanisms and cooling technology of PEMFCs. The research results proved that the liquid metal technique, radiative cooling technique, and spray cooling system could effectively improve the cooling performance of the thermal management system. Song [8] analyzed the effects of temperature on the performance of fuel cell vehicles’ energy management strategies. Then, they analyzed the effects of temperature on the PEMFCs. Su [9] proposed a decoupled control strategy and built a controller of the pump and fan. Then, they established a model of a fuel cell cooling system. Analysis results showed that a decoupled control strategy could improve the heat dissipation efficiency of the cooling system. He [10] analyzed cooling methods and the control strategy of PEMFCs’ thermal management systems. Comparing the characteristics of different control strategies. The investigation results indicated that reasonable cooling methods and control strategies are essential to maintain the stable operation of PEMFCs. Cao [11] proposed a kind of low-temperature thermal management system, which combines phase change materials and liquid cooling techniques, analyzing temperature variations in PEMFCs under different conditions. The research results indicated that a proper loop design can improve the temperature uniformity.

Although many scholars have investigated control strategies and cooling methods of a Proton Exchange Membrane Fuel Cell, the low efficiency of heat dissipation remains a big challenge. Liquid cooling technology has been the most dominant heat dissipation method for Proton Exchange Membrane Fuel Cells. However, the insufficient heat dissipation capacity of existing coolants limits the application of high-power fuel cell technology [12]. In 2004, a nano-coolant was applied as a coolant in fuel cells by the U.S. Department of Energy. The research results proved that the nano-coolant could improve heat dissipation performance effectively [13].

### 1.2. Nano-Coolant Overview

**Figure 3:**
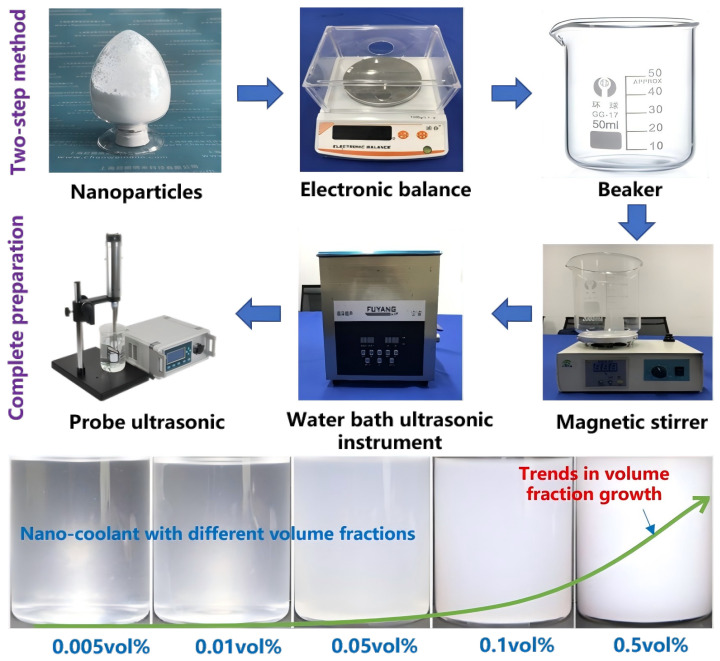
Nano-coolant preparation method.

A nano-coolant is a new type of two-phase coolant that is mixed with solid particles and a base coolant. It has a higher thermal conductivity and better heat transfer capacity than a traditional single-phase coolant. Many researchers have delved into nano-coolants in PEMFCs. For example, Bargal [14] summarized the literature about liquid cooling techniques in the PEFMCs and proved that the nano-coolant is a promising substitution to replace the existing coolant. Islam [15] explored the heat dissipation capacity of ZnO nano-coolant as a coolant in 2.4 kW PEMFCs. The research results indicated that the radiator’s dimension was decreased by 25% with a 2 vol% ZnO nano-coolant. Islam [16] also analyzed the thermophysical properties of the TiO_2_ nano-coolant at a volume fraction 0.05~0.5 vol% via theoretical analysis and experimental tests. The test results showed that heat transfer performance was enhanced by 10% with a 0.5 vol% TiO_2_ nano-coolant. Zakaria [17] investigated the heat transfer performance enhancement of PEMFCs using 0.1 vol% and 0.5 vol% Al_2_O_3_ nano-coolants. The effect of different flow rates (Reynolds number in 20~120) for heat transfer performance was analyzed. Compared with base fluid, the heat dissipation power of 0.5 vol% Al_2_O_3_ nano-coolant was increased by 13.87%. Zakaria [18] analyzed the heat dissipation capacity of an Al_2_O_3_ nano-coolant with different volume fractions. The experimental results proved that the heat dissipation capacity of the 0.1 vol% Al_2_O_3_ nano-coolant with a base fluid ratio of 60:40 is better than 50:50. The preparation method of the nano-coolant can be seen in Figure 3 below.

The research above is mainly based on metal oxide nanoparticles. Some scholars investigated nano-coolants of nonmetallic nanoparticles mixed with a base fluid. In addition, many researchers mixed two or more nanoparticles to prepare a multi-mixed nano-coolant and investigated its heat dissipation performance in a PEMFC [19,20,21]. Based on the previous research above, nano-coolant applications in PEMFCs are summarized in Table 1 below.

**Figure 4:**
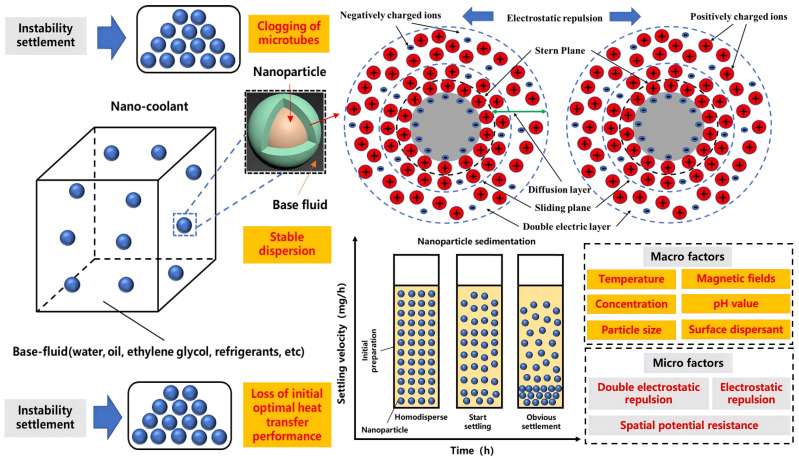
Schematic diagram of nano-coolant destabilization and sedimentation [12].

According to the theory of electrostatic stabilization (Derjaguin Landau Vewey Overbeek), due to the different environmental factors, nanoparticles inside the nano-coolant are settled by van der Waals’ gravitational force and universal gravitational force, which causes clogging in the pipeline. It also affects the initial heat transfer performance and thermophysical properties of the nano-coolant. The main influencing factors include temperature, particle size, concentration, pH value, etc. [22]. This can be seen in Figure 4. Hence, it is important to delve into the methods of a nano-coolant’s stability improvement.

### 1.3. Thermal Conductivity of Nano-Coolant

At present, many researchers and scientists have proposed different classical theoretical models of solid–liquid mixtures’ thermal conductivity [12]. However, these thermal conductivity models are developed based on the theory of the practical medium, which assumes nanoparticles remain fixed and discontinuous in base fluid. These models are unsuitable for solid–liquid mixtures, which neglect the process of heat transfer from the nanoparticle to base fluid. The impacting factors of thermal conductivity for nano-coolants include the dimension and shape of nanoparticles, volume fraction of the nano-coolant, temperature of the nano-coolant, etc. [23]. The Maxwell model is the earliest thermal conductivity model that can be used to calculate the thermal conductivity of liquid–solid mixtures with low volume fractions and spherical nanoparticles [24]. The Maxwell equation is as follows:

(1) $$\frac{k_{nf}}{k_{bf}}=\frac{k_{p}+2k_{bf}+2\left(k_{p}-k_{bf}\right)\varphi }{k_{p}+2k_{bf}-2\left(k_{p}-k_{bf}\right)\varphi }$$

k_nf_ is the nano-coolant’s thermal conductivity, k_p_ is the nanoparticle’s thermal conductivity, φ is the nano-coolant’s volume fraction, and k_bf_ is the base fluid’s thermal conductivity.

Bruggeman [25] proposed the thermal conductivity model of a suspension fluid, which can be used to calculate the thermal conductivity of liquid–solid mixtures with spherical particles. The equation is as follows:

(2) $$k_{nf}=\frac{1}{4}\left[3\left(\varphi -1\right)\frac{k_{p}}{k_{bf}}+\left(2-3\varphi \right)+\sqrt{\Delta }\right]$$

(3) $$\Delta =\left[3{\left(\varphi -1\right)}^{2}{\left(k_{p}/k_{b}\right)}^{2}+{\left(2-3\varphi \right)}^{2}+2\left(2+9\varphi -9\varphi^{2}\right)\left(k_{p}/k_{b}\right)\right]$$

k_nf_ is the nano-coolant’s thermal conductivity, k_p_ is the nanoparticle’s thermal conductivity, φ is the nano-coolant’s volume fraction, and k_bf_ is the base fluid’s thermal conductivity.

Hamilton and Crosser also proposed a type of thermal conductivity model, which considers the nanoparticle’s sphericity [26].

(4) $$\frac{k_{nf}}{k_{bf}}=\frac{k_{p}+\left(n-1\right)k_{bf}+\left(n-1\right)\left(k_{p}-k_{bf}\right)\varphi }{k_{p}+\left(n-1\right)k_{bf}-\left(k_{p}-k_{bf}\right)\varphi }$$

n is the nanoparticles’ sphericity

(5) n=3/ψ

Yamada and Ota developed a type of thermal conductivity mode by using the parameter k to instead of (n − 1) in Hamilton and Crosser’s model [27].

(6) $$\frac{k_{nf}}{k_{bf}}=\frac{k_{p}+kk_{bf}+k\left(k_{p}-k_{bf}\right)\varphi }{k_{p}+kk_{bf}-\left(k_{p}-k_{bf}\right)\varphi }$$

k_nf_ is the nano-coolant’s thermal conductivity, k_p_ is the nanoparticle’s thermal conductivity, φ is the nano-coolant’s volume fraction, and k_bf_ is the base fluid’s thermal conductivity.

(7) $$k=2\varphi^{-0.2}$$

If the nanoparticle is cylindrical, the parameter k can be expressed as follows:

(8) $$k=2\varphi^{0.2}\frac{L}{d}$$

L is the particle’s length, and d is the particle’s diameter.

In addition, Davis also proposed a type of thermal conductivity model, which is only valid for the nano-coolant with low volume fractions and spherical nanoparticles [28].

(9) $$\frac{k_{nf}}{k_{bf}}=1+\frac{3\left(1-\alpha \right)\varphi }{\left(1+2\alpha \right)-\left(1-\alpha \right)\varphi }\left[\varphi +f\left(\alpha \right)\varphi^{2}+O\left(\varphi^{3}\right)\right]$$

(10) $$f\left(\alpha \right)=\sum_{p=6}^{\infty }\left[\left(B_{p}-3A_{p}\right)/\left(p-3\right)2^{p-3}\right]$$

A_p_ and B_p_ are the constants related to the parameters α and p.

Although there are many different thermal conductivity models proposed, there is no unified opinion on the mechanism of the micro thermal conductivity of the nano-coolant. Hence, it is essential to delve into the mechanism of a nano-coolant’s thermal conductivity.

### 1.4. Brief Summary

In order to more accurately calculate the actual heat dissipation of the thermal management system for PEMFCs, it is significant to develop an accurate theoretical model of thermal conductivity of a nano-coolant. Although many theoretical models have been proposed, these models neglected the process of heat transfer from the nanoparticle to base fluid. Hence, this paper delved into the mechanism of a nano-coolant’s thermal conductivity.

## 2. Thermal Conductivity Model Formulation

### 2.1. Theoretical Analysis

**Figure 5:**
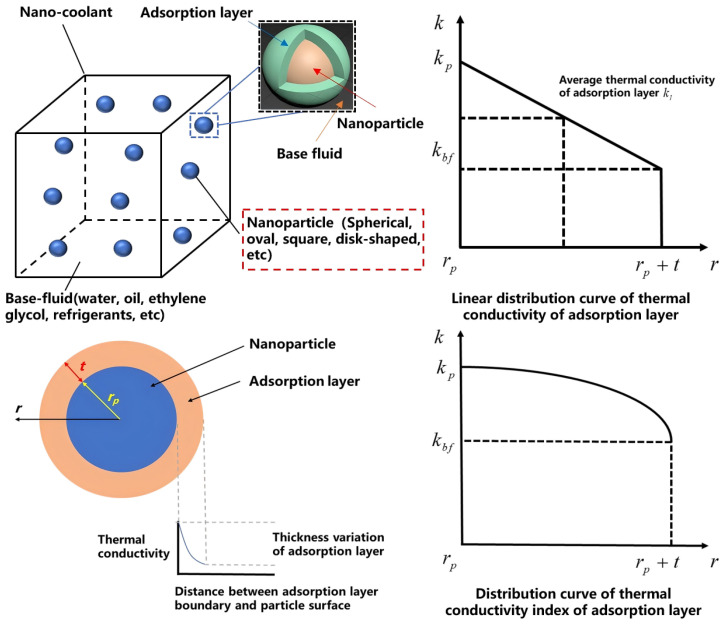
Schematic diagram of nanoparticles and adsorption layer [12].

In recent years, many researchers proposed three kinds of thermal conductivity mechanisms of nano-coolants from a microcosmic perspective. It includes Brownian motion, the adsorption layer, and nanoparticle aggregation. But it still cannot reach an agreement on enhancing the thermal conductivity of a nano-coolant [29]. Many scholars coupled two or three mechanisms. However, the mechanism of Brownian motion is gradually weakened. There are many scholars who supported the adsorption layer and particle agglomeration mechanism on the particle surface to explain the thermal conductivity enhancement for nano-coolants [30]. This can be seen in Figure 5 below [31,32].

According to the Figure 5 above, heat could be transferred from nanoparticles to the base fluid. However, the thermal conductivity of a nano-coolant is significantly influenced by the nanolayer thickness, which is not a constant value but varies with the volume fraction, radius of nanoparticles, and nano-coolant temperature. Nanolayer thickness is proposed by scientists, which is used to analyze the nano-coolant thermal conductivity. It is not easy to measure in practice. Hence, obtaining the adsorption layer thickness via theoretical analysis is general [33]. For example, Li, et al. [34] analyzed the effect of the volume fraction of nanoparticles, nanoparticle radius, and the temperature on the adsorption layer thickness of a nano-coolant. Then, they proposed an empirical correlation of the adsorption layer thickness. Finally, they developed a novel semi-empirical model for predicting the thermal conductivity of an oil-based nano-coolant.

Many researchers also investigated the thermal conductivity of the adsorption layer for nano-coolants. For example, Jiang [35] established a cubed distribution curve of thermal conductivity for nano-coolants’ adsorption layers, which is close to the physical reality. Research results indicated that adsorption layer thickness and nanoparticle diameter have an obvious influence on the effective thermal conductivity of nano-coolants. Xie [36] analyzed and proposed linear distribution law of nano-coolants’ thermal conductivity with the adsorption layer theory. The equation of an adsorption layer’s thermal conductivity distribution curve is shown as follows:

(11) $$k_{l}(r)=\frac{k_{bf}-k_{p}}{t}r+\frac{k_{bf}(r_{p}+t)-k_{bf}r_{p}}{t}$$

k_l_ (r) is the thermal conductivity of the adsorption layer, k_bf_ is the thermal conductivity of base fluid, k_p_ is the thermal conductivity of nanoparticles, r_p_ is the radius of nanoparticle, and t is the thickness of the adsorption layer.

According to the linear distribution curve in Figure 5, the average thermal conductivity of the adsorption layer in a nano-coolant can be seen in the following equation:

(12) $$k_{l}=\frac{k_{p}+k_{bf}}{2}$$

According to the analysis of thermal resistance, Xie also investigated the average thermal conductivity of a nano-coolant with a spherical nanoparticle under linear distribution [36].

(13) $$\frac{k_{l}}{k_{bf}}=\frac{1}{\alpha }\frac{{\left(1+\beta -\alpha \right)}^{2}}{\left(1+\beta -\alpha -\alpha \beta \right)In\left(1+\beta /\alpha \right)+\beta \left(1+\beta -\alpha \right)}$$

k_l_ is the average thermal conductivity of a nano-coolant’s adsorption layer, and α and β are dimensionless parameters, which can be defined as follows:

(14) $$\alpha =k_{bf}/k_{p}\;\beta =t/r_{p}$$

Some scholars also delved into a linear distribution model of the adsorption layer thermal conductivity [37,38,39]. Moreover, some scholars investigated the nonlinear law of adsorption layer thermal conductivity. For example, Tillman [40] proposed an exponential distribution curve for the average thermal conductivity of a nano-coolant’s adsorption layer, which is expressed below:

(15) $$k_{t}\left(r\right)=k_{0}{\left(1-Ar\right)}^{m}\;k_{t}\left(r\right)=k_{0}{\left(1-A/r\right)}^{m}\;k_{t}\left(r\right)=k_{0}e^{-\frac{A}{r^{m}}}$$

where k_0_ and A are coefficients, and m is an exponential coefficient.

Xue [40] also developed an effective thermal conductivity model of nano-coolants based on the theory of practical medium and the theory of average polarization, considering the effects of the adsorption layer and nanoparticle geometry.

(16) $$k_{pe}=k_{l}\frac{k_{p}+2k_{l}+\frac{2(k_{p}-k_{l})}{{\left(1+\beta \right)}^{3}}}{k_{p}+2k_{l}-\frac{k_{p}-k_{l}}{{\left(1+\beta \right)}^{3}}}$$

k_pe_ is the thermal conductivity, which combines the nanoparticle and adsorption layer; k_p_ is the thermal conductivity of nanoparticles; k_l_ is the average thermal conductivity of a nano-coolant’s adsorption layer; and β is the ratio of adsorption layer thickness and the nanoparticle radius. The nanoparticle volume fraction changes when combined with the thermal conductivity of adsorption layer and nano-particles.

(17) $$\varphi_{e}={\left(1+\beta \right)}^{3}\varphi$$

However, the Hamilton & Crosser equation is the classical thermal conductivity model, which is considered the effects of nanoparticle geometry. Hence, this paper proposed a novel model of nano-coolant’s thermal conductivity, which synthesized the Xue model and Hamilton & Crosser model. In addition, to adapt proton exchange membrane fuel cells, the experimental data of a base fluid’s thermal conductivity are measured and used to fit a model suitable for PEMFCs.

### 2.2. Micromorphology Analysis of Nanoparticles

**Figure 6:**
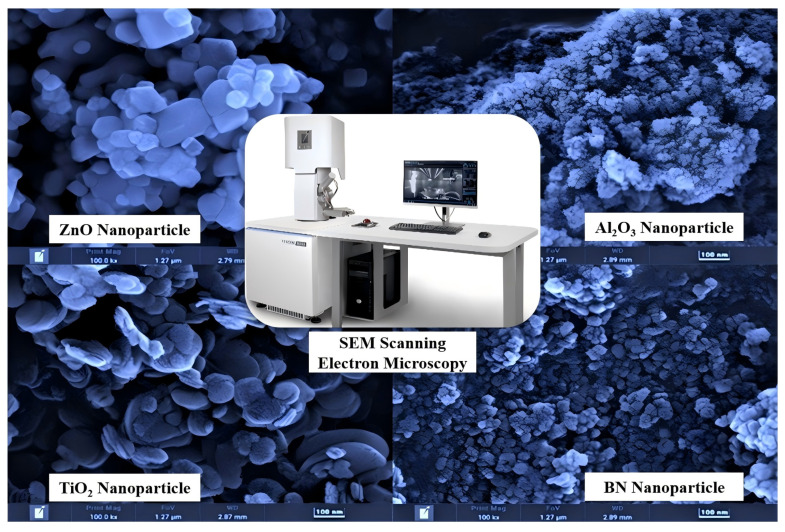

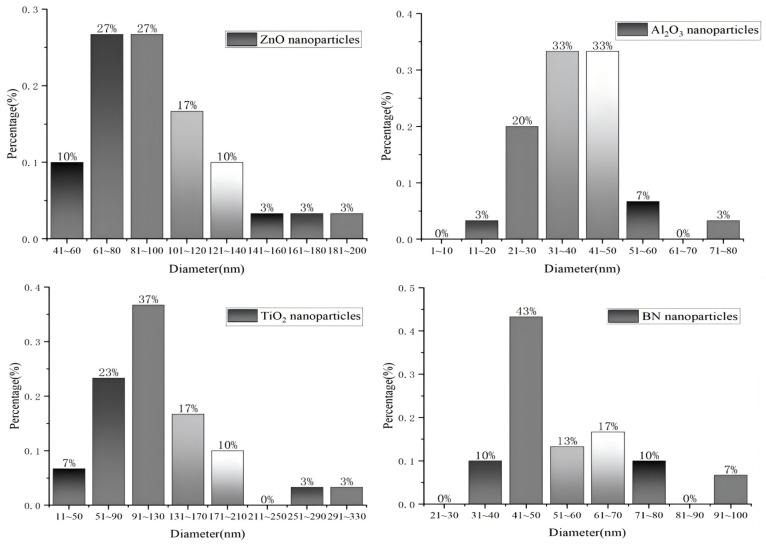
Microstructures of ZnO, Al_2_O_3_, TiO_2_, and BN nanoparticles.

In order to analyze the geometry, dimension, and distribution of different nanoparticles, an SEM field emission scanning electron microscope (Tescan Mira4, TESCAN Group a.s., Shanghai, China) was used to analyze the microscopic morphology of nanoparticles. Firstly, conductive tape or liquid conductive adhesive was used to fix nanoparticles on the sample table and mark, and the sequence number of nanoparticles is marked on the sample table. Then, the samples were sprayed with gold and dried after treatment. After that, the sample table was adjusted to a suitable height, and the sample table was put into the sample chamber of the SEM field emission scanning electron microscope. Adjust the observation height of the equipment, and observe the samples with the sequence number. Finally, remove the sample table, and turn off the equipment. The microscopic morphologies of zinc oxide, alumina, titanium oxide, and boron nitride nanoparticles were measured using SEM scanning electron microscopy. The testing result can be seen in Figure 6.

The geometry of the ZnO nanoparticle is approximately cuboid, the geometries of Al_2_O_3_ and TiO_2_ nanoparticles are approximately spherical, and the geometry of the BN nanoparticle is approximately disc-shaped. Figure 6 above shows that the dimensions of four nanoparticles are 60 nm, 30 nm, 50 nm, and 80 nm, respectively. According to the degree of sphericity data of Table 2, the degrees of sphericity of ZnO, Al_2_O_3_, TiO_2_, and BN nanoparticles are 0.77, 1.0, 1.0, and 0.58, respectively [41].

### 2.3. Thermophysical Property Comparison of Different Nano-Coolants

Thermophysical properties of nano-coolants include the thermal conductivity, density, specific heat, and convective heat transfer coefficient. The density of the nano-coolant can be calculated using the equation below [43]:

(18) $$\rho_{nf}=(1-\varphi )\rho_{bf}+\varphi \rho_{p}$$

ρ_nf_ is the density of the nano-coolant, ρ_nf_ is the density of the base fluid, ρ_p_ is the density of the nanoparticle, and φ is the volume fraction of the nano-coolant.

The specific heat of the nano-coolant can be calculated as in the equation below [43]:

(19) $$C_{nf}=(1-\varphi )C_{bf}+\varphi C_{p}$$

C_nf_ is the specific heat of the nano-coolant, C_bf_ is the specific heat of the base fluid, C_p_ is the specific heat of the nanoparticle, and φ is the volume fraction of the nano-coolant.

Nano-coolant thermal conductivity can be calculated based on Hamilton & Crosser, as the following equation [43]:

(20) $$\frac{k_{nf}}{k_{bf}}=\frac{k_{p}+\left(n-1\right)k_{bf}+\left(n-1\right)\left(k_{p}-k_{bf}\right)\varphi }{k_{p}+\left(n-1\right)k_{bf}-\left(k_{p}-k_{bf}\right)\varphi }$$

The nano-coolant convective heat transfer coefficient can be calculated as the equation below [43]:

(21) $$K=\frac{1}{\frac{1}{h_{i}}+R_{i}+\frac{\delta }{\lambda }+\frac{A_{i}}{A_{o}}\frac{1}{h_{o}}+\frac{A_{i}}{A_{o}}R_{o}}$$

K is the convective heat transfer coefficient, h_i_ and h_0_ are the convective heat transfer coefficients inside and outside the flat tube, R_i_ and R_0_ are the fouling resistance inside and outside the flat tube, A_i_ and A_0_ are the heat exchange area inside and outside the flat tube, δ is the thickness of the flat tube, and λ is the thermal conductivity of the flat tube.

### 2.4. Thermal Conductivity Model for PEMFC

At present, the existing coolant of fuel cell vehicles blends ethylene glycol and deionized water in a proportion. Compared with the traditional oil-fueled vehicles, the density, specific heat, freezing point, boiling point, corrosion resistance, electrical conductivity, and thermal conductivity of the coolant are different. This is based on the thermal conductivity model of Hamilton & Crosser, combined with the adsorption layer and the parameter of standard fuel cell vehicle’s cooling medium. A novel theoretical model of nano-coolant thermal conductivity suitable for Proton Exchange Membrane Fuel Cells can be established. Thermophysical parameters of the coolant for PEMFCs are shown in Table 3 [44].

According to the coolant standard of fuel cell vehicles, the freezing point should be ≤−35 °C. Based on the data of Table 3, if the proportion of ethylene glycol and deionized water is >48%, the freezing point requirement can be achieved. Therefore, this paper used base fluid parameters with volume fractions of 50%, 55%, and 60%, which are shown in Table 4 below. This can be used to establish a novel thermal conductivity model of fuel cell vehicle coolant with the quadratic function.

Volume fraction 50%:

(22) $$Y=-4.4\times 10^{-6}x^{2}+9.536\times 10^{-4}x+0.3629$$

Volume fraction 55%:

(23) $$Y=-4.55\times 10^{-6}x^{2}+9.4275\times 10^{-4}x+0.3491$$

Volume fraction 60%:

(24) $$Y=-3.83\times 10^{-6}x^{2}+8.2582\times 10^{-4}x+0.3346$$

**Figure 7:**
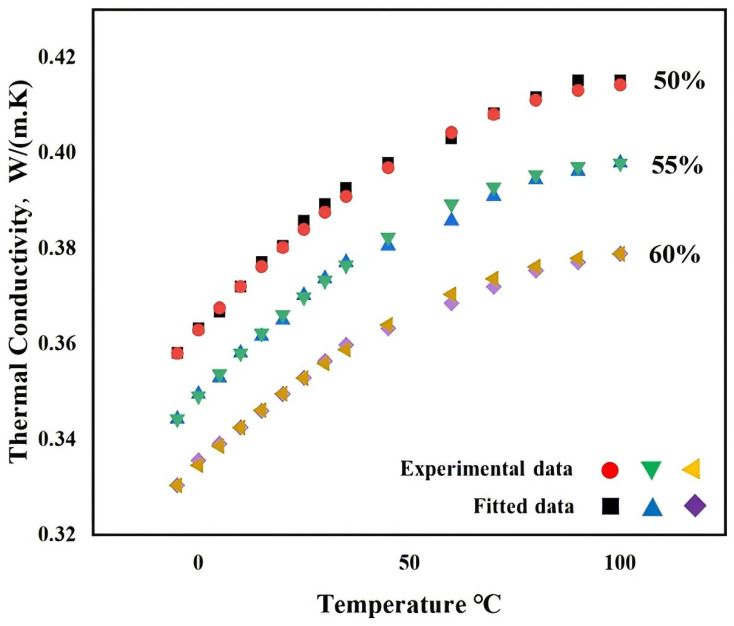
Experimental data and fitted data of base fluid’s thermal conductivity.

The value x is ranged from −5 °C to 100 °C, and the base fluid thermal conductivity at different temperatures and volume fractions are shown in Figure 7.

According to Figure 7, the thermal conductivity of different volume fractions of the base fluid is increased with an increase in the base fluid’s temperature. The thermal conductivity of the base liquid can be calculated by using Equations (12)–(14). The analysis results indicated that theoretical data can be matched with the actual thermal conductivity of the base fluid. When the temperature is the same, the thermal conductivity difference for a volume fraction of 50~55% is close to the volume fraction of 55~60%. With the increase in the base fluid’s temperature, the thermal conductivity difference between the volume fraction of 50~55% and 55~60% is increased gradually. The equation for the thermal conductivity difference between the volume fraction of 50~55% and 55~60% for the base fluid is shown in the equation below.

(25) $$\Delta k=-6.9388\times 10^{-7}T^{2}+1.1449\times 10^{-4}T+0.01449$$

Considering the nano-coolant adsorption layer theory, the thermal conductivity of a nano-coolant’s adsorption layer can be defined as the average value of the nanoparticle and base fluid. In addition, the composite nanoparticle volume fraction can be calculated by the following:

(26) $$\varphi_{e}={\left(1+\beta \right)}^{3}\varphi$$

Finally, a new model of nano-coolant thermal conductivity suitable for PEMFCs was obtained, which can be seen in the following Equations (18)–(20):

(27) $$k_{nf}=\frac{k_{pe}+\left(n-1\right)k_{bf}\left(T\right)+\left(n-1\right)\left[k_{pe}-k_{bf}\left(T\right)\right]\varphi_{e}}{k_{pe}+\left(n-1\right)k_{bf}\left(T\right)-\left[k_{pe}-k_{bf}\left(T\right)\right]\varphi_{e}}k_{bf}\left(T\right)$$

k_nf_ is the nano-coolant’s thermal conductivity, k_pe_ is the composite nanoparticles’ thermal conductivity, n is the degree of sphericity of the nanoparticle, and T is the temperature of the base fluid. The temperature range is −35~100 °C, k_bf_ is the thermal conductivity of the base fluid, and φ_e_ is the volume fraction of composite nanoparticles.

(28) $$k_{pe}=k_{l}\frac{k_{p}+2k_{l}+2\left(k_{p}-k_{l}\right)/{\left(1+\beta \right)}^{3}}{k_{p}+2k_{l}-\left(k_{p}-k_{l}\right)/{\left(1+\beta \right)}^{3}}$$

k_pe_ is the composite nanoparticles’ thermal conductivity, and k_l_ is the thermal conductivity of the adsorption layer. k_p_ is the nanoparticles’ thermal conductivity. β is the ratio of the adsorption layer and nanoparticle size.

A few researchers researched the adsorption layer thickness because it is currently impossible to obtain its size accurately with an experimental test. In this paper, the thickness of the adsorption layer is assumed to be 0.5 nm.

(29) $$k_{bf}\left(T\right)=-4.4\times 10^{-6}T^{2}+9.536\times 10^{-4}T+0.3629-\frac{\left(\varphi_{bf}-0.5\right)}{0.05}\times \Delta k$$

k_bf_ is the base fluid thermal conductivity, T is the temperature of the base fluid, the temperature range is −35~100 °C, φ_bf_ is the volume fraction of the composite nanoparticles (>50%), and ∆_k_ is the base fluid difference.

(30) $$\Delta k=-6.9388\times 10^{-7}T^{2}+1.1449\times 10^{-4}T+0.01449$$

where ∆_k_ is the difference value for the base fluid, and T is the temperature of the base fluid.

## 3. Conclusions

A nano-coolant is a promising coolant, which has great potential to replace existing coolants of PEMFCs. Although there are three thermal conductivity mechanisms of nano-coolants, which have been proposed in recent years, specifically Brownian motion, adsorption layer, nanoparticle aggregation, it still cannot reach an agreement on the enhancing of thermal conductivity for a nano-coolant. This study established a new type of thermal conductivity model, which is suitable for PEMFCs. The main conclusions of this study are as follows:

(1) Analyzing the type of fuel cells, as well as the application objects of Proton Exchange Membrane Fuel Cells. Then introducing the structure of a thermal management system of Proton Exchange Membrane Fuel Cells and the current problems. Thus, the nano-coolant is proposed.
(2) According to the previous research of nano-coolant applications in PEMFCs, ZnO, Al_2_O_3_, TiO_2_, and BN nano-coolants were selected to delve into the thermal conductivity model of nano-coolants suitable for PEMFCs. The mechanism of nano-coolant settling was also analyzed.
(3) Comparing and analyzing the classical thermal conductivity model of a nano-coolant, which were proposed in recent years. Focusing on the theory of nano-coolants’ adsorption layer thermal conductivity, which include linear and non-linear distribution curves.
(4) The microscopic characterization of nanoparticles was obtained via SEM scanning electron microscopy. The experimental results could be used to obtain the sphericity of different nanoparticles based on the microscopic morphology measurement.
(5) Combining the Hamilton & Crosser model and nano-coolants’ adsorption layer theory, a new thermal conductivity model of nano-coolants suitable for PEMFCs was established. This model can be used to calculate the thermal conductivity of nano-coolants in PEMFCs.

## Figures and Tables

**Table 1:** Nano-coolant application in PEMFC [14,15,16,17,18,19,20,21].

Author	Research/Time	Base Fluid	Nanoparticle	Volume Fraction
Zakaria [17]	Theory/2015	W/EG 50:50	Al_2_O_3_	0.1 vol%/0.5 vol%
Zakaria [17]	Experiment/2015	W/EG 50:50	Al_2_O_3_	0.1 vol%/0.5 vol%
Bargal [14]	Theory/2020	W/EG 50:50	Al_2_O_3_	0.05 vol%/2 vol%
Zakaria [18]	Experiment/2016	W/EG 50:50	Al_2_O_3_	0.1 vol%/0.5 vol%
		W/EG 60:40		
Islam [15]	Theory/Experiment 2017	W/EG 50:50	TiO_2_	0.05 vol%~0.5 vol%
Islam [15]	Experiment/2017	W/EG 50:50	ZnO	0.05 vol%~0.5 vol%
Zakaria [18]	Theory/2018	W/EG 100:0	Al_2_O_3_	0.1 vol%~0.5 vol%
		W/EG 50:50		
		W/EG 60:40		
Zakaria [18]	Experiment/2018	W/EG 100:0 W/EG 60:40	Al_2_O_3_	0.1 vol%~0.5 vol%
Ilhan [19]	Experiment/2016	W/EG 50:50	BN	0.03 vol%~3 vol%
Johari [20]	Experiment/2022	W/EG 60:40	Al_2_O_3_-SiO_2_	0.5 vol%
Khalid [21]	Experiment/2021	W/EG 60:40	Al_2_O_3_-SiO_2_	0.5 vol%

**Table 2:** Nanoparticle sphericity [42].

Nanoparticle Shape		Sphericity	Nanoparticle Shape		Sphericity
Spherical		1.0	Regular tetrahedron		0.67
Cylinder	H = d	0.87	Regular octahedron		0.83
	H = 2d	0.83	Cuboid	1:2:2	0.77
	H = 4d	0.73		1:2:4	0.68
Disc	H = d/2	0.83		1:4:4	0.64
	H = d/4	0.69	Ellipsoid	1:1:2	0.93
	H = d/10	0.58		1:1:4	0.78
Cuboid	1:1:1	0.81		1:2:2	0.92
	1:1:2	0.77		1:2:4	0.79
	1:1:4	0.68		1:4:4	0.70

**Table 3:** Freezing point and density of coolant for PEMFCs [44].

Volume Fraction %	Freezing Point °C	Density	Volume Fraction %	Freezing Point °C	Density
46	−32.2	1.068	54	−43.9	1.078
47	−33.9	1.069	55	−45.6	1.081
48	−35.0	1.070	56	−46.7	1.082
49	−36.1	1.072	57	−47.8	1.083
50	−37.8	1.073	58	−48.9	1.084
51	−38.9	1.074	59	<−51.1	1.085
52	−41.1	1.076	60	<−51.1	1.086
53	−42.2	1.077	65	<−51.1	1.093

**Table 4:** Thermal conductivity of coolant for PEMFCs [44].

T (°C)	50%	55%	60%
−5	0.3581	0.3443	0.3304
0	0.3633	0.3495	0.3356
5	0.3668	0.3529	0.3391
10	0.3720	0.3581	0.3425
15	0.3771	0.3616	0.3460
20	0.3806	0.3650	0.3495
25	0.3858	0.3702	0.3529
30	0.3893	0.3737	0.3564
35	0.3927	0.3771	0.3598
45	0.3979	0.3806	0.3633
60	0.4031	0.3858	0.3685
70	0.4083	0.3910	0.3720
80	0.4117	0.3944	0.3754
90	0.4152	0.3962	0.3771
100	0.4152	0.3979	0.3789

## Data Availability

Data are contained within the article.

## Nomenclature

Abbreviations	
PEMFC	Proton Exchange Membrane Fuel Cell
PAFC	Phosphoric Acid Fuel Cell
SOFC	Solid Oxide Fuel Cell
MCFC	Molten Carbonate Fuel Cell
SOFC	Solid Oxide Fuel Cell
AFC	Alkaline Fuel Cell
U.S.	United States
ZnO	Zinc Oxide
TiO_2_	Titanium Oxide
Al_2_O_3_	Aluminum Oxide
BN	Boron Nitride
Al_2_O_3_-SiO_2_	Aluminum Oxide- Silicon Dioxide
DLVO	Derjaguin Landau Vewey Overbeek
SEM	Scanning Electron Microscope
Symbols	
$\rho_{nf}$	Nano-coolant’s density
$\rho_{bf}$	Base fluid’s density
$\rho_{p}$	Nanoparticle’s density
φ	Nano-coolant’s volume fraction
*C_nf_*	Nano-coolant’s specific heat
*C_bf_*	Base fluid’s specific heat
*C_p_*	Nanoparticle’s specific heat
*k_nf_*	Nano-coolant’s thermal conductivity
*k_bf_*	Base fluid’s thermal conductivity
*k_p_*	Nanoparticle’s thermal conductivity
n	Shape factor
Ψ	Sphericity degree
*K*	Convective heat transfer coefficient
*h_i_*	Convective heat transfer coefficient inside flat tube
*h_0_*	Convective heat transfer coefficient outside flat tube
*R_i_*	Fouling resistance inside flat tube
*R_0_*	Fouling resistance outside flat tube
*A_i_*	Heat exchange area inside flat tube
*A_0_*	Heat exchange area outside flat tube
δ	Thickness of flat tube
λ	Flat tube thermal conductivity
L	Nanoparticle length
d	Nanoparticle diameter
*k_l_*	Average thermal conductivity of Nano-coolant adsorption layer
